# A Potential Role for Bile Acid Signaling in Celiac Disease-Associated Fatty Liver

**DOI:** 10.3390/metabo12020130

**Published:** 2022-01-30

**Authors:** Paul Manka, Svenja Sydor, Julia M. Schänzer-Ocklenburg, Malte Brandenburg, Jan Best, Ramiro Vilchez-Vargas, Alexander Link, Dominik Heider, Susanne Brodesser, Anja Figge, Andreas Jähnert, Jason D. Coombes, Francisco Javier Cubero, Alisan Kahraman, Moon-Sung Kim, Julia Kälsch, Sonja Kinner, Klaas Nico Faber, Han Moshage, Guido Gerken, Wing-Kin Syn, Ali Canbay, Lars P. Bechmann

**Affiliations:** 1 Department of Internal Medicine, University Hospital Knappschaftskrankenhaus, Ruhr-University Bochum, In der Schornau 23-25, 44892 Bochum, Germany; paul.manka@rub.de (P.M.); svenja.sydor@rub.de (S.S.); jan.best@kk-bochum.de (J.B.); anja.figge@kk-bochum.de (A.F.); andreas.jaehnert@ruhr-uni-bochum.de (A.J.); ali.canbay@rub.de (A.C.); 2 Department of Gastroenterology and Hepatology, University Hospital Essen, Hufelandstrasse 55, 45147 Essen, Germany; julia.schaenzer@arcor.de (J.M.S.-O.); brandenburgetlam@gmail.com (M.B.); alisan.kahraman@uk-essen.de (A.K.); julia.kaelsch@uk-essen.de (J.K.); guido.gerken@helios-gesundheit.de (G.G.); 3 Department of Gastroenterology, Hepatology, and Infectious Diseases, Otto-von-Guericke-University Hospital Magdeburg, Leipziger Strasse 44, 39120 Magdeburg, Germany; ramiro.vilchez@med.ovgu.de (R.V.-V.); Alexander.Link@med.ovgu.de (A.L.); 4 Department of Mathematics and Computer Science, Philipps-University Marburg, Hans-Meerwein-Straße 6, 35043 Marburg, Germany; heiderd@informatik.uni-marburg.de; 5 Cluster of Excellence Cellular Stress Response in Aging-associated Diseases (CECAD) Faculty of Medicine, University Hospital of Cologne, University of Cologne, Joseph-Stelzmann-Str. 26, 50931 Cologne, Germany; susanne.brodesser@uk-koeln.de; 6 Inflammation Biology, Faculty of Life Sciences and Medicine, King’s College London, London WC1E6H, UK; jason.d.coombes@kcl.ac.uk; 7 Department of Immunology, Opthalmology and ENT, Complutense University School of Medicine, 28040 Madrid, Spain; fcubero@ucm.es; 8 Centro de Investigación Biomédica en Red de Enfermedades Hepáticas y Digestivas (CIBEREHD), Instituto de Salud Carlos III, 28220 Madrid, Spain; 9 Instituto de Investigación Sanitaria Gregorio Marañón (IiSGM), 28007 Madrid, Spain; 10 Department of Diagnostic and Interventional Radiology and Neuroradiology, University Hospital Essen, Hufelandstrasse 55, 45147 Essen, Germany; moon-sung.kim@uk-essen.de (M.-S.K.); sonja.kinner@uk-essen.de (S.K.); 11 University Medical Center Groningen, Department of Gastroenterology and Hepatology, University of Groningen, Hanzeplein 1, 9713 GZ Groningen, The Netherlands; k.n.faber@umcg.nl (K.N.F.); a.j.moshage@umcg.nl (H.M.); 12 University Medical Center Groningen, Department of Laboratory Medicine, University of Groningen, Hanzeplein 1, 9713 GZ Groningen, The Netherlands; 13 Division of Gastroenterology and Hepatology, Department of Medicine, Medical University of South Carolina, Charleston, SC 29425, USA; synw@musc.edu; 14 Section of Gastroenterology, Ralph H. Johnson Veterans Affairs Medical Center, Charleston, SC 29425, USA; 15 Department of Physiology, Faculty of Medicine and Nursing, University of Basque Country UPV/EHU, 489040 Vizcaya, Spain

**Keywords:** celiac disease, hepatic steatosis, bile acids, FGF19, non-alcoholic fatty liver disease (NAFLD)

## Abstract

Celiac disease (CeD) is a chronic autoimmune disorder characterized by an intolerance to storage proteins of many grains. CeD is frequently associated with liver damage and steatosis. Bile acid (BA) signaling has been identified as an important mediator in gut–liver interaction and the pathogenesis of non-alcoholic fatty liver disease (NAFLD). Here, we aimed to analyze BA signaling and liver injury in CeD patients. Therefore, we analyzed data of 20 CeD patients on a gluten-free diet compared to 20 healthy controls (HC). We furthermore analyzed transaminase levels, markers of cell death, BA, and fatty acid metabolism. Hepatic steatosis was determined via transient elastography, by MRI and non-invasive scores. In CeD, we observed an increase of the apoptosis marker M30 and more hepatic steatosis as compared to HC. Fibroblast growth factor 19 (FGF19) was repressed in CeD, while low levels were associated with steatosis, especially in patients with high levels of anti-tissue transglutaminase antibodies (anti-tTG). When comparing anti-tTG-positive CeD patients to individuals without detectable anti-tTG levels, hepatic steatosis was accentuated. CeD patients with significant sonographic steatosis (defined by CAP ≥ 283 db/m) were exclusively anti-tTG-positive. In summary, our results suggest that even in CeD patients in clinical remission under gluten-free diet, alterations in gut–liver axis, especially BA signaling, might contribute to steatotic liver injury and should be further addressed in future studies and clinical practice.

## 1. Introduction

Celiac disease (CeD) is a chronic autoimmune disorder characterized by a lifelong intolerance to the storage of proteins of many grains. The disease can manifest with both intestinal and extraintestinal symptoms. The worldwide prevalence is approximately 0.5–1%, with significant regional variations [1]. Undiagnosed CeD patients are assumed to account for a significant number of cases, given the high prevalence of asymptomatic courses and the heterogeneous manifestation. CeD is frequently associated with other autoimmune diseases such as Hashimoto’s thyroiditis or type I diabetes mellitus [2]. Recently, liver injury emerged as a rare but serious extraintestinal manifestation of CeD. The spectrum ranges from simple steatosis with or without hepatic inflammation, autoimmune hepatitis, to liver failure [3,4,5,6]. Accordingly, several guidelines implemented screening for CeD in patients with fatty liver disease [7,8].

However, the mechanisms of CeD-associated steatosis and liver injury are not entirely understood. The interaction of gut and liver via enterohepatic circulation might be a potential link in the pathogenesis of CeD-associated steatosis. Primary bile acids are produced from cholesterol in hepatocytes via cholesterol-7-alpha-hydroxylase (CYP7A1) and enter the bile. CYP7A1 expression is regulated by the farnesoid X receptor (FXR), which is activated via bile acids in a negative feedback-loop. FXR and its hepatic target gene NR0B2 control the small heterodimer partner and the intestinal expression of fibroblast growth factor 19 (FGF19), jointly regulating the de novo synthesis of primary bile acids [9]. In the intestine, some bile acids are modified by various bacteria to secondary bile acids, reabsorbed from the ileum, and returned to the liver via the portal bloodstream. We and others have previously identified alterations in bile acid metabolism and gut–liver interactions as pivotal players in the pathogenesis of hepatic steatosis and its complications [10,11,12]. Similarly, alterations in bile acid homeostasis, enterohepatic bile acid pool, and gut microbiota composition have been described in CeD [13,14]. However, little is known about these interrelations in CeD patients in clinical remission. 

Therefore, we aimed to investigate the role of the gut–liver axis with a focus on bile acid metabolism, hepatic steatosis, and liver damage in a CeD cohort in clinical remission by following a gluten-free diet. 

## 2. Results

### 2.1. Demographic Data

The control group (*n* = 20) included 3 males and 17 females aged 22 to 60 years (median 29.5). The CeD group (*n* = 20) consisted of 1 male and 19 females aged 20 to 57 years (median 30). 

Since CeD is associated with a change in diet and malnutrition may also be present, the subjects were evaluated for their nutritional status. There were no significant differences between the groups concerning BMI and fat mass determined using BIA measurement. Similarly, HDL, cholesterol, triglycerides, and HbA1c did not differ significantly (Table 1).

**Figure 1:**
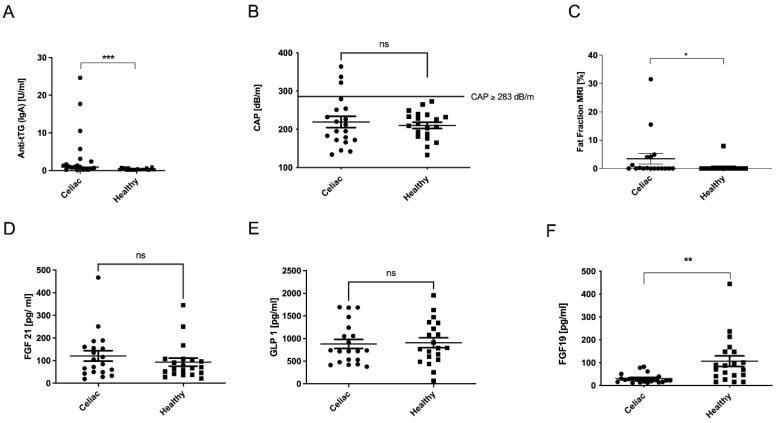
A trend toward hepatic steatosis in patients with CeD. Despite gluten-free diets, celiac patients had elevated levels of anti-tissue transglutaminase antibodies (anti-tTG) (**A**). In the celiac group, patients showed higher CAP levels than controls, and in addition, there were more individuals in the celiac group who had CAP levels significantly above the threshold of 283 dB/m (**B**). Celiac patients also had a higher fat content in the liver measurable by MRI (**C**). Serum concentrations of the incretins FGF21 (**D**) and GLP1 (**E**) did not differ significantly between groups, and FGF19 serum levels were decreased in CeD patients (**F**). * *p* < 0.05, ** *p* < 0.01, *** *p* < 0.001; ns stands for not significant.

Liver synthesis parameters, platelet count, and INR were examined but did not show significant differences between control and CeD (Table 1). While at the time of inclusion into our study, all CeD patients were in clinical remission on a gluten-free diet for many years, few CeD patients had detectable levels of tissue transglutaminase antibodies (anti-tTG), which were significantly increased compared to the control group (Figure 1A).

### 2.2. Celiac Disease and Non-Invasive Liver Tests

We performed several non-invasive tests to assess hepatic steatosis, fibrogenic remodeling of the liver tissue, and other liver-related differences between subjects and controls. To assess fibrogenic remodeling of the liver tissue, we performed transient elastography measurement (TEM), as well as measurement of the controlled attenuation parameter (CAP) to determine steatosis. 

There were no differences in CAP between the two groups. However, in contrast to healthy controls, in the CeD group, we identified individuals who had a CAP value consistent with hepatic steatosis (Figure 1B), as defined by a cut-off value of ≥283 db/m [15,16]. In contrast, magnetic resonance imaging (MRI) examination revealed significantly more steatosis in the CeD group compared to controls (Figure 1C). The degree of fibrosis did not seem to differ (Table 1). Although there were no differences in transaminase levels (Table 1), the CeD group expressed higher serum levels of the apoptosis marker M30 than healthy controls, indicating hepatocellular apoptosis as a sign of liver injury (Table 1). However, the cell death marker M65 did not vary between the groups (Table 1).

Inflammatory processes and immunologic responses related to gut permeability can be triggered in the context of CeD. Therefore, the serum concentrations of the inflammatory serum marker lipoprotein binding protein 1 (LBP1) and calprotectin in feces were measured. Here, there were no differences in the parameters in comparison between the groups (Table 1).

### 2.3. Incretin Levels in Celiac Disease

The gut–liver axis refers to the interaction between the gut and the liver via the enterohepatic circulation of intestinal hormones (also known as incretins). Together with bile acids and related target genes, gut-derived cytokines, bacterial components, and other gut-derived factors, these hormones affect several processes in the liver. Serum levels of other incretins such as fibroblast growth factor 21 (FGF21) and glucagone-like peptide 1 (GLP1) did not differ between the two groups (Figure 1D,E). Interestingly, serum levels of the bile acids’ regulated FXR target gene FGF19 were significantly suppressed in CeD patients’ serum compared to controls, despite the majority of the CeD patients being in clinical remission (Figure 1F). 

**Figure 2:**
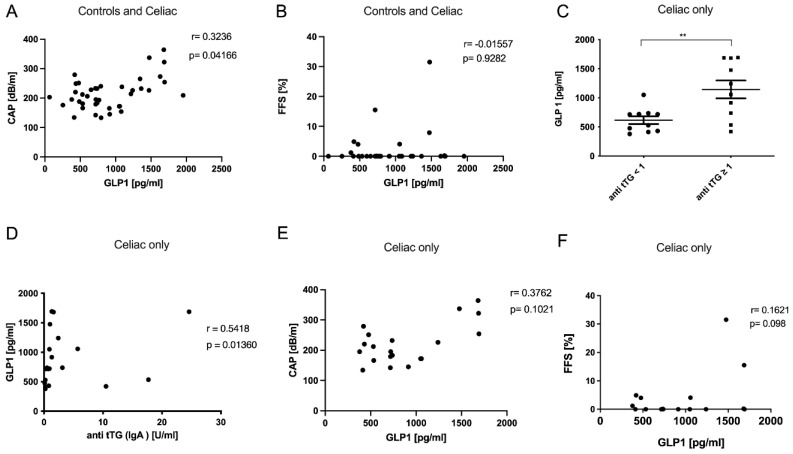
GLP1 serum levels were associated with increased hepatic steatosis. The degree of steatosis as assessed by CAP (**A**) was associated with GLP1 in the entire study cohort, while this could not be confirmed by MRI (**B**). Celiac disease patients with increased anti-tTG antibodies had higher GLP1 serum concentrations (**C**), and these parameters also showed a positive correlation (**D**). Looking at the association of GLP1 with steatosis only in the celiac patients, we found the same trend by both CAP (**E**) and MRI measurements (**F**). ** *p* < 0.01.

The degree of steatosis assessed by CAP was associated with GLP1 in the study cohort, while this interrelation could not be confirmed by fat fraction measurement via MRI (Figure 2A,B). However, GLP1 was increased in CeD patients with higher levels of transglutaminase antibodies and these two parameters also showed a positive correlation (Figure 2C,D). Considering the CeD group only, we confirmed a trend towards a positive association of GLP1 with the degree of steatosis (Figure 2E,F).

### 2.4. Bile Acid Metabolism in Celiac Disease

Serum levels of total bile acids were not changed when comparing the two groups (Table 1). On the basis of the specific role of FGF19 in bile acid metabolism, we examined the composition of individual primary and secondary unconjugated and conjugated bile acids in both serum and feces. However, we observed significantly higher levels of the secondary conjugated bile acid GLCA in serum of CeD patients (Table S1). Levels of individual bile acids in fecal samples did not differ between groups (Table S2). Interestingly, FGF19 levels were suppressed in CeD patients’ serum compared to the controls (Figure 1F). 

**Figure 3:**
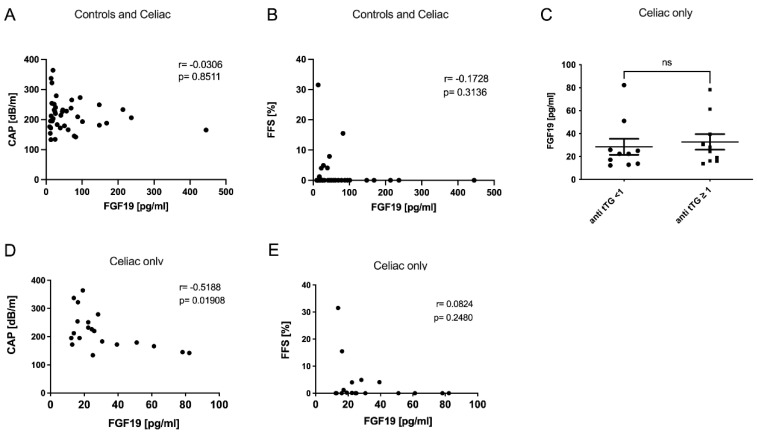
FGF19 serum levels of CeD patients were associated with increased hepatic steatosis. Neither CAP (**A**) nor fat fraction as assessed by MRI (**B**) was significantly associated with FGF19 levels in the entire cohort. Comparing FGF19 serum levels in celiac patients with different anti-tTG antibodies levels, we found that no differences between the groups were detected (**C**). However, when comparing the association of FGF19 with steatosis in the celiac patients only, CAP showed a significant correlation with FGF19 (**D**). Correlation of FGF19 with MRI measurement was not significant (**E**). ns stands for not significant.

Correlation analyses revealed no association of FGF19 with liver injury or steatosis parameters in the total cohort (Figure 3A,B and Figure S1). However, while there was no difference comparing FGF19 levels regarding the levels of transglutaminase antibodies, there was a clear negative correlation of FGF19 with CAP within the CeD cohort alone (Figure 3C–E). We additionally assessed several non-invasive scores that may be applied to evaluate liver injury. In detail, we calculated the FibroScan-AST (FAST) [17] and the modified CheK score (Chek_mod_), as previously published by our group [18]. The CheK_mod_ score in particular significantly associated with CAP, FGF19, and FGF21 (Figure S1E–G) within the CeD group.

### 2.5. FGF-19 Levels in Celiac Disease Were Dependent on Disease Activity

**Figure 4:**
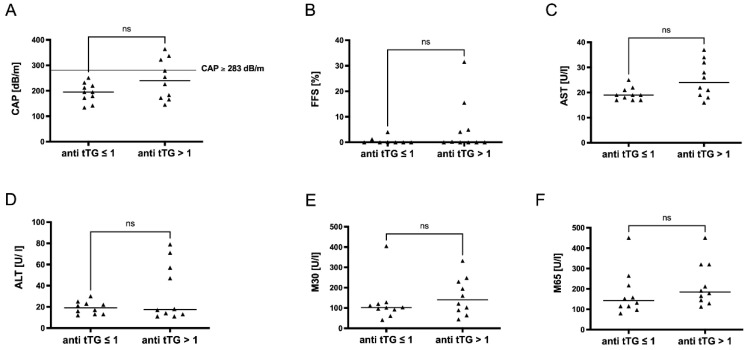
A subgroup analysis showed trended differences in steatotic liver injury depending on disease activity. In a subgroup cohort analysis comparing celiac patients with anti-tTG levels ≤ 1 and those with anti-tTG levels > 1, we could show that those patients with anti-tTG > 1 showed a trend towards increased steatosis as assessed by CAP (**A**) and MRI-FFS (**B**). Those individuals with anti-tTG levels > 1 showed a tendency of increased transaminase levels of AST (**C**) and ALT (**D**) as well as of hepatocellular apoptosis marker M30 (**E**) and overall cell death marker M65 (**F**). ns stands for not significant.

**Figure 5:**
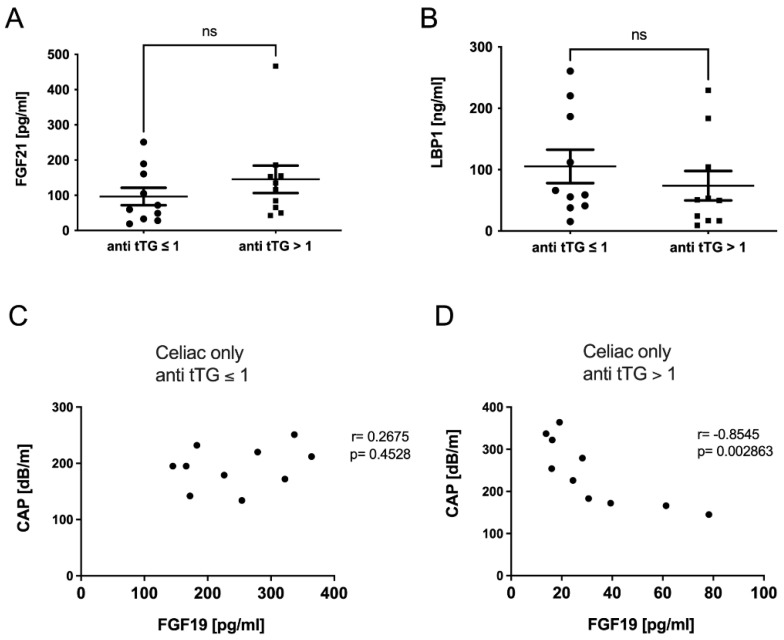
FGF19 serum levels in celiac disease were associated with disease activity. The incretins FGF21 (**A**) and LBP1 (**B**) did not differ between patients with anti-tTG ≤ 1 and anti-tTG > 1. FGF19 levels in CeD patients with antitTG ≤ 1 showed no significant correlation with CAP (**C**), but an invert association with steatosis as assessed by CAP was seen for CeD patients with antitTG > 1 (**D**). ns stands for not significant.

Due to differences in CeD activity within the cohort, we performed a subgroup analysis comparing patients with detectable serum levels of anti-tTG to those without detectable anti-tTG levels. Patients with anti-tTG ≥ 1 U/mL showed a trend towards increased steatosis that was not significant (Figure 4A,B). As described above, in steatotic CeD patients with CAP ≥ 283 db/m (Figure 1B), anti-tTG were above 1 U/mL. A similar trend was observed for other markers of hepatocellular injury (Figure 4C–F). The incretins FGF21 and LBP1 were slightly increased in individuals but did not show any significant differences either (Figure 5A,B), while GLP1 levels were significantly increased in anti-tTG ≥ 1 U/mL (Figure 2C). 

Correlation analyses within the subgroups indicated that patients with transglutaminase antibody levels ≥1 U/mL present with an invert association between FGF19 and the extent of steatosis as assessed by CAP (Figure 5D). Interestingly, we did not observe this association in patients with antibody levels <1 U/L (Figure 5C), while in patients with ant tTG > 1 U/L, this association was significant (Figure 5D). Similarly, markers of liver cell damage tended to associate negatively with FGF19 levels without reaching statistical significance (Figure S2A–D). 

### 2.6. Intestinal Motility Correlated with FGF19 in Celiac Disease

**Figure 6:**
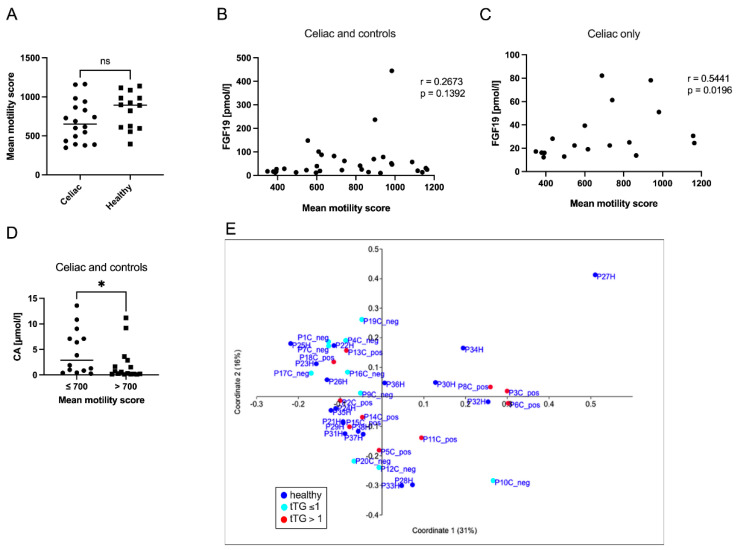
Intestinal motility correlated with FGF19 in celiac disease. Intestinal motility was measured by MRI and showed a tendency of lower motility in celiac patients (**A**). FGF19 showed a positive trend without statistical significance when correlated with intestinal motility in the whole cohort (**B**). When analyzing the celiac patients only, we found that FGF19 and measurement of intestinal motility correlated significantly (**C**). Levels of the primary bile acids CA (**D**) were compared in the whole cohort, comparing those individuals with a slower (≤700 mean motility score) versus patients with a faster gut motility (>700 mean motility score). Microbiome analysis based on 16sDNA sequencing of fecal samples showed no clear delineation of specific groupings when comparing control, anti-tTG-positive (anti-tTG > 1), and anti-tTG-negative (anti-tTG < 1) individuals (**E**). * *p* < 0.05, ns stands for not significant.

Using MRI measurement, we examined the intestinal motility of the subjects. Celiac patients presented lower intestinal motility than control subjects, but this difference was not significant (Figure 6A). Parameters of steatosis or liver injury did not correlate with the intestinal motility (data not shown). When FGF19 was correlated with intestinal motility, a positive trend was seen for the entire cohort (controls and CeD, Figure 6B). This trend turned to a clear positive association between levels of FGF19 and intestinal motility when analyzing CeD patients only (Figure 6C). In individuals (CeD and controls) with slower gut motility (≤700 versus >700 mean motility score), serum levels of the primary bile acid CA were significantly increased (Figure 6D). Furthermore, when comparing only CeD patients, serum levels of CDCA, FGF19, and CAP were also changed in relation to gut motility but did not reach significance (Figure S2E–H). 

We were also interested in whether there are shifts within the gut microbiome comparing controls and CeD patients. Therefore, we performed a 16s DNA microbiome analysis from fecal samples. However, initial studies showed that the individual samples from controls and CeD patients (including discrimination between tTG-positive and tTG-negative individuals) could not be distinguished from each other (Figure 6E). 

## 3. Discussion

In this study, we aimed to elucidate gut–liver interaction in CeD-related steatosis and liver injury in a cohort of CeD patients in clinical remission. In our cohort, FGF19 was repressed in CeD. Low levels of FGF19 were associated with hepatic steatosis, especially in patients with high levels of anti-tTG antibodies. 

Some liver abnormalities in CeD, including asymptomatic elevations of transaminases, hepatic steatosis, nonspecific hepatitis, autoimmune hepatitis, and cholestatic liver diseases, may occur primarily in the active period [19]. It has previously been shown that NAFLD patients have increased intestinal permeability and that steatosis is associated with small intestinal bacterial overgrowth (SIBO) [20,21]. Higher intestinal permeability and increased zonulin levels have been found to correlate with the level of steatosis. Lipotoxic molecules (e.g., cholesterol), LPS, and other harmful substances can thus more easily pass the intestinal barrier, leading to the activation of various signaling cascades, resulting in apoptosis, oxidative stress, inflammation, and disruption of mitochondrial function. These findings suggest that bacterial translocation may be related to increased gut permeability and steatosis. In this context, after the diagnosis and under a gluten-free diet, the course of hepatic steatosis and hepatic inflammation may improve, as the intestinal barrier can rebuild and metabolic processes are normalized [21]. In our study, the microbiome analysis did not reveal any relevant differences compared to the control group. However, since all patients were on a gluten-free diet, these results may be biased. We also assessed SIBO and *H. pylori* status in all study participants using standard breath tests. In the cohort, two controls and one CeD patient showed a SIBO-positive test (Table 1), but these individuals did not show strikingly elevated levels of steatosis parameters or the other parameters of liver injury. All participants had a negative breath test for *H. pylori*.

Contrarily, after the onset of gluten-free diet, the risk of developing NAFLD increases 4–6-fold, especially within the first five years [22]. Various causes have been discussed thus far: On the one hand, prolonged malabsorption can lead to altered lipid metabolism in mitochondria. This consequently leads to reduced β-oxidation and increased fat storage in hepatocytes [23]. Secondly, untreated CeD patients usually have a lower body weight than healthy individuals due to disturbed absorption. After starting a gluten-free diet, weight normalization usually occurs. However, gluten-free products often have higher fat, salt, and carbohydrate content and lower fiber content than conventional products. Therefore, the gluten-free diet carries the risk of obesity, dyslipidemia, and metabolic syndrome with the possible development of steatosis [24,25].

Intestinal FGF19 physiologically represses hepatic lipogenesis. Hepatic farnesoid X receptor (FXR) protein level and circulating FGF19 concentration is low in children with NAFLD [26]. To what extent CeD-induced enterocyte damage leads to dysregulation of FGF19 remains unclear. CeD may lead to dysregulation in the terminal ileum. In this context, it would be conceivable that a reduced FGF19 production occurs and leads to less negative feedback and increased bile acid synthesis, including its toxic metabolites. In our studies, no change in the bile acid profile could be demonstrated. In this context, it should be mentioned that the maladaptation is not necessarily in the bowel system as CeD is a systemic disorder. Ijssennagger et al. demonstrated in an animal model that a dysregulation of the hepatic FXR leads to a significant metabolic dysregulation, whereas the enteral restrictions showed less influence [27]. Interestingly, we observed a clear association of FGF19 and gut motility as assessed by MRI. Serum levels of primary bile acids such as CA and CDCA were higher in individuals with slow motility compared to those with fast motility. In brief, we assume that gut motility affects the clearance and effects of the intestinal bile acid pool and its activation of intestinal FXR, as previously shown in BA-induced diarrhea [28]. Again, none of the CeD patients suffered from diarrhea in our cohort, and given the fact that we recruited adults with CeD in clinical remission under gluten-free diet, it might be an expectable result that total bile acid levels were not changed.

Our study does have some limitations. The size of the cohort is relatively small. Further, all patients were in clinical remission and followed a gluten-free diet, which may somewhat mitigate our statements regarding the condition of an uncontrolled or untreated CeD, as observance of a gluten-free diet has previously been identified as a strong predictor of liver injury in CeD patients [29]. 

Nonetheless, we provide strong evidence that in the context of CeD, the gut–liver axis and, in particular, the FGF19-FXR pathway may play an essential role in pathological hepatic processes. Evidence that FGF19 levels remain suppressed in patients despite their gluten-free diet should give reason to investigate this issue for a possible pathological role in follow-up studies.

## 4. Methods 

### 4.1. Ethical Statement and Sample Collection

Patients were prospectively recruited in the Department of Gastroenterology and Hepatology at Essen University Hospital from September 2016 until April 2017. The study was approved by the Essen University Hospital Ethics committee (Institutional Review Board; reference number: 14-6044-BO), and the study protocol followed the ethical guidelines of the Declaration of Helsinki. All subjects provided informed written consent. 

Diagnosis of CeD was based on the fulfillment of diagnostic criteria at the time when individual patients were diagnosed [30]. A total of 7 of the 20 celiac disease subjects had been diagnosed within five years before the study was conducted. For the remaining participants, the diagnosis was established more than 5 years ago. All subjects reported strict adherence to the gluten-free diet since diagnosis and did not report any symptoms at time of inclusion into the study. Controls were stated as healthy and did not show any known intestinal or liver-related diseases. None of the control subjects followed a gluten-free diet or adhered to any specific dietary pattern. Since celiac disease is associated with a change in diet and malnutrition may also be present, all subjects were examined for their nutritional status using a general questionnaire also asking for present intestinal complaints. There were no significant differences between the groups regarding BMI and fat mass determined using BIA measurement.

Serum samples were collected in a fasted state and stored in aliquots at −80 °C until for analysis. Patients’ fecal samples were collected in sterile tubes and stored at −80 °C until further analysis. Standard laboratory parameters were evaluated via the central laboratory of the Essen University Hospital. 

To determine colonization with *H. pylori*, we performed an established ^13^C-urea breath test in order to exclude SIBO we performed an established H_2_-glucose breath test. All breath tests were performed and in the fasting state. 

### 4.2. ELISA

Serum levels of hepatocellular apoptosis marker M30, overall cell death marker M65 and adiponectin were measured using commercially available kits from TecoMedical (Sissach, Switzerland). Serum levels of fibroblast growth factor 19 (FGF19) and FGF21 were quantified using the Quantikine ELISA kit purchased from R&D Systems (Minneapolis, MN, USA) and levels of glucagon-like peptide 1 (GLP1) by using the GLP1 ELISA kit from Abcam (Cambridge, United Kingdom). Lipopolysaccharide-binding protein (LBP), which is a marker for intestinal inflammation, was quantified with the human LBP ELISA kit from Hycult Biotech (Uden, the Netherlands). All ELISA kits were performed according to the manufacturer’s instructions. The concentrations of fecal calprotectin to evaluate inflammatory processes of the intestinal mucosa were measured using the BÜHLMANN fCal ELISA Kit (Bühlmann Laboratories AG, Schönenbuch, Switzerland). 

### 4.3. Serum and Fecal Sample Bile Acid Profiling

Fecal bile acids were extracted by sonification in 1:3 diluted extraction buffer (ethanol and phosphate buffer, Sigma-Aldrich, Steinheim, Germany) from approximately 300 mg of feces; supernatants were used for quantification. Quantification of primary and secondary bile acids from serum and fecal sample extracts was performed by liquid chromatography coupled to electrospray ionization tandem mass spectrometry (LC–ESI–MS/MS) using the Biocrates^®^ Bile Acids kit (BIOCRATES Life Science AG, Innsbruck, Austria), which covers 16 individual bile acids. Data analysis was completed using the Biocrates MetIDQ software (Version MetIDQ 7.11.5-DB180-Nitrogen-2834) [31].

### 4.4. Assessment of Liver Steatosis via Transient Elastography

We combined TEM via Fibroscan^®^ with the CAP to measure hepatic fat accumulation. We defined steatosis following a cut-off value of CAP ≥ 283 db/m, which was described previously for determining fatty liver [15,16].

### 4.5. Bioelectrical Impedance Analysis (BIA)

Bioelectrical impedance analysis was performed in order to measure the distribution of water, fat, and muscle in the body using the BIACORPUS RX 4000 (MEDI CAL HealthCare GmbH, Karlsruhe, Germany). According to the so-called “three-compartment model”, fat mass (kg and %), body cell mass, and extracellular mass (kg) were distinguished. Extracellular and body cell mass together form the fat-free mass (kg). The analysis of the measurement data was performed, taking into account gender, age, height, and weight by the software BodyComp V 8.5 (MEDI CAL HealthCare GmbH).

### 4.6. Magnetic Resonance Imaging (MRI) for Liver Fat Detection and Bowel Movement Analysis

MRI of the bowel and liver was performed on a 3 Tesla magnet (Magnetom Avanto, Siemens Health Care, Erlangen, Germany). Conventional axial in and opposed phase (IOP) imaging of the liver was performed, and average values for fat and water images were measured using regions of interest (ROI). After that, fat signal fractions can be calculated, as shown by Reeder et al. [32]. Coronal real-time true-Fisp sequences of the bowel were acquired for automatic generation of parametric maps facilitating quantification of bowel motility as previously reported [33].

### 4.7. Statistical Analysis

Statistical significance was determined using the Mann–Whitney *U* test. Correlation analysis was performed using linear regression analysis; all analyses were performed using GraphPad Prism 9. If not stated otherwise, all data are presented as means ± SEM, and statistical significance was assumed at *p* ≤ 0.05.

## Figures and Tables

**Table 1:** Overview of demographic data of the study cohort. * *p* < 0.05. n.s. stands for not significant.

	Controls (*n* = 20) 17 Female/3 Male	Celiac Disease (*n* = 20) 19 Female/1 Male	*p*-Value
**Age**	29.5 (median)	30.0 (median)	n.s.
**SIBO-positive**	2/20	1/20	n.s.
**Body mass index (kg/m^2^)**	23.13 ± 4.60	23.22 ± 0.94	n.s.
**TEM (kPa)**	5.65 ± 0.59	4.39 ± 0.29	n.s.
**Body Fat Mass BIA (%)**	18.14 ± 9.62	19.74 ± 8.93	n.s.
**Total bile acids (µmol/L)**	4.43 ± 1.09	3.26 ± 0.04	n.s.
**Cholesterol (mg/dl)**	188.10 ± 39.50	194.80 ± 52.08	n.s.
**HDL (mg/dl)**	71.75 ± 18.58	62.95 ± 12.66	n.s.
**Triglycerides (mg/dl)**	79.50 ± 39.91	82.00 ± 37.01	n.s.
**HbA1c (%)**	5.33 ± 0.24	5.30 ± 0.34	n.s.
**Platelets (n/dl)**	240.50 ± 45.79	243.00 ± 56.59	n.s.
**INR**	0.99 ± 0.04	0.99 ± 0.05	n.s.
**ALT (U/L)**	19.30 ± 1.27	26.50 ± 4.57	n.s.
**AST (U/L)**	22.50 ± 2.53	22.35 ± 1.37	n.s.
**Alkaline phosphatase (U/L)**	60.25 ± 17.62	57.75 ± 13.19	n.s.
**Bilirubin (mg/dl)**	0.68 ± 0.32	0.63 ± 0.34	n.s.
**γGT (U/L)**	13.90 ± 7.35	18.30 ± 22.02	n.s.
**LDH (U/L)**	181.10 ± 51.28	168.10 ± 20.11	n.s.
**GLDH (U/L)**	2.81 ± 2.80	3.30 ± 2.35	n.s.
**M30 (U/L)**	88.91 ± 6.00	142.40 ± 21.40	*
**M65 (U/L)**	169.30 ± 14.18	200.40 ± 24.34	n.s.
**LBP1 (ng/mL)**	89.25 ± 16.05	89.66 ± 18.05	n.s
**Calprotectin, feces (µg/g)**	40.49 ± 4.99	52.60 ± 7.77	n.s

## Data Availability

The data presented in this study are available on request from the corresponding author. The data are not publicly available due to privacy and ethical reasons.

## Supplementary Materials

The following supporting information can be downloaded at: https://www.mdpi.com/article/10.3390/metabo12020130/s1, Figure S1: Correlation analysis of FGF19 with parameters of liver injury and hepatic steatosis in the whole study cohort, Figure S2: Correlation analysis of FGF19 with parameters of liver injury in CeD patients, Table S1: Concentrations of individual serum bile acids, Table S2: Concentrations of individual fecal bile acids.
