# Supplementary material for: A Potential Role for Bile Acid Signaling in Celiac Disease-Associated Fatty Liver
Source: Metabolites. 2022 Jan 30;12(2):130. doi: 10.3390/metabo12020130 (PMC8879761; doi:10.3390/metabo12020130)
Supplement: Supplementary file 1 [file metabolites-12-00130-s001.zip › metabolites-1555160-supplementary.pdf]

Supplementary Material to the Manuscript:

A potential role for bile acid signaling in celiac disease-associated fatty liver.

Manka P, Sydor S, Schänzer-Ocklenburg JM, Brandenburg M, Best J, Vilchez-Vargas R, Link A, Heider D, Brodesser S, Figge A, Jähnert A, Coombes JD, Cubero FJ, Kahraman A, Kim MS, Kälsch J, Kinner S, Faber KN, Moshage H, Gerken G, Syn WK, Canbay A, Bechmann LP

Supplementary Table S1: Concentrations of individual serum bile acids. * = $p<0.05$

**Serum concentration [µmol/l]**	**Healthy (n=20)**	**Celiac disease (n=20)**	**P value**
**Primary unconjugated bile acids**			
Cholic acid (CA)	2.23 ± 0.65	4.23 ± 1.23	n.s.
Chenodeoxycholic acid (CDCA)	2.82 ± 0.71	4.31 ± 1.18	n.s.
**Primary conjugated bile acids**			
Glycocholic acid (GCA)	2.98 ± 0.46	3.01 ± 0.52	n.s.
Taurocholic acid (TCA)	0.62 ± 0.15	0.39 ± 0.08	n.s.
Glycochenodeoxycholic acid (GCDCA)	11.22 ± 1.77	13.57 ± 2.64	n.s.
Taurochenodeoxycholic acid (TCDCA)	2.22 ± 0.51	1.51 ± 0.30	n.s.

**Secondary unconjugated bile acids**			
Deoxycholic acid (DCA)	7.41 ± 1.23	6.72 ± 0.82	n.s.
Lithocholic acid (LCA)	0.13 ± 0.05	0.14 ± 0.04	n.s.
Ursodeoxycholic acid (UDCA)	1.03 ± 0.37	1.33 ± 0.26	n.s.
**Secondary conjugated bile acids**			
Glycodeoxycholic acid (GDCA)	4.84 ± 0.86	6.08 ± 1.55	n.s.
Taurodeoxycholic acid (TDCA)	0.96 ± 0.21	0.71 ± 0.17	n.s.
Glycolithocholic acid (GLCA)	0.17 ± 0.03	0.28 ± 0.06	*
Glycoursodeoxycholic acid (GUDCA)	1.68 ± 0.61	2.20 ± 0.61	n.s.
Taurolithocholic acid (TLCA)	0.06 ± 0.01	0.07 ± 0.01	n.s.
Tauroursodeoxycholic acid (TUDCA)	0.12 ± 0.03	0.11 ± 0.02	n.s.

Supplementary Table S2: Concentrations of individual fecal bile acids.

**Feces concentration [µmol/g]**	**Healthy (n=20)**	**Celiac disease (n=20**	**P value**
**Primary conjugated bile acids**			
Cholic acid (CA)	0.27 ± 0.1	0.54 ± 0.44	n.s.
Chenodeoxycholic acid (CDCA)	0.32 ± 0.12	0.66 ± 0.41	n.s.

**Primary conjugated bile acids**			
Glycocholic acid (GCA)	0.01 ± 0.003	0.01 ± 0.01	n.s.
Taurocholic acid (TCA)	0.01 ± 0.01	0.01 ± 0.01	n.s.
Glycochenodeoxycholic acid (GCDCA)	0.02 ± 0.01	0.02 ± 0.01	n.s.
Taurochenodeoxycholic acid (TCDCA)	0.004 ± 0.001	0.01 ± 0.004	n.s.
**Secondary unconjugated bile acids**			
Deoxycholic acid (DCA)	8.51 ± 1.00	8.43 ± 1.02	n.s.
Lithocholic acid (LCA)	7.39 ± 0.90	6.83 ± 0.96	n.s.
Ursodeoxycholic acid (UDCA)	0.17 ± 0.08	0.27 ± 0.13	n.s.
**Secondary conjugated bile acids**			
Glycodeoxycholic acid (GDCA)	0.03 ± 0.01	0.04 ± 0.01	n.s.
Taurodeoxycholic acid (TDCA)	0.01 ± 0.01	0.4 ± 0.02	n.s.
Glycolithocholic acid (GLCA)	0.01 ± 0.003	0.01 ± 0.002	n.s.
Glycoursodeoxycholic acid (GUDCA)	0.002 ± 0.001	0.004 ± 0.002	n.s.
Taurolithocholic acid (TLCA)	0.01 ± 0.01	0.02 ± 0.01	n.s.
Tauroursodeoxycholic acid (TUDCA)	0.0003 ± 0.0001	0.001 ± 0.0005	n.s.

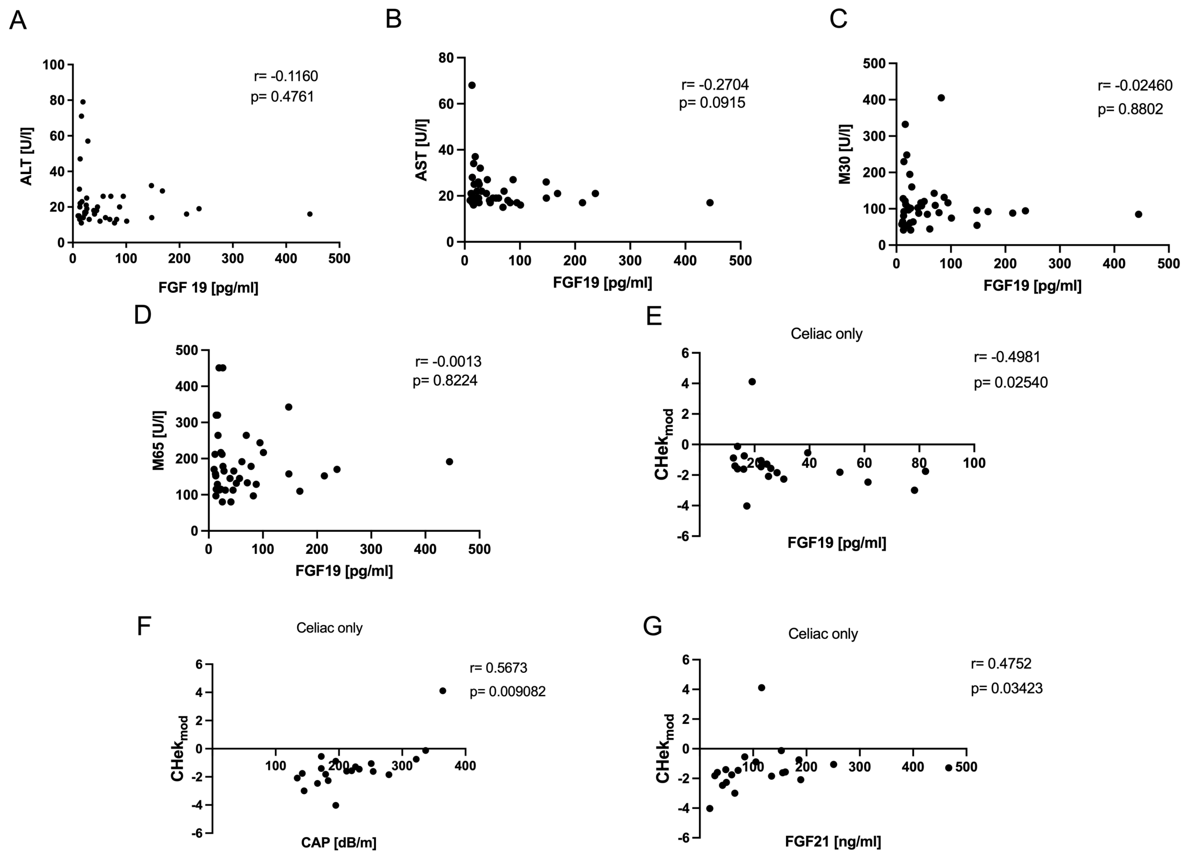

Supplementary Figure S1: Correlation analysis of FGF19 with parameters of liver injury and hepatic steatosis in the whole study cohort. Serum FGF19 levels were not significantly correlated with liver injury markers ALT (A), AST (B), M30 (C) and M65 (D) in the whole study cohort (CeD and healthy controls). The CheK$_{mod}$, which is a predictive score describing liver injury correlated with FGF19 (E), CAP (F) and FGF21 (G) in the celiac patients only.

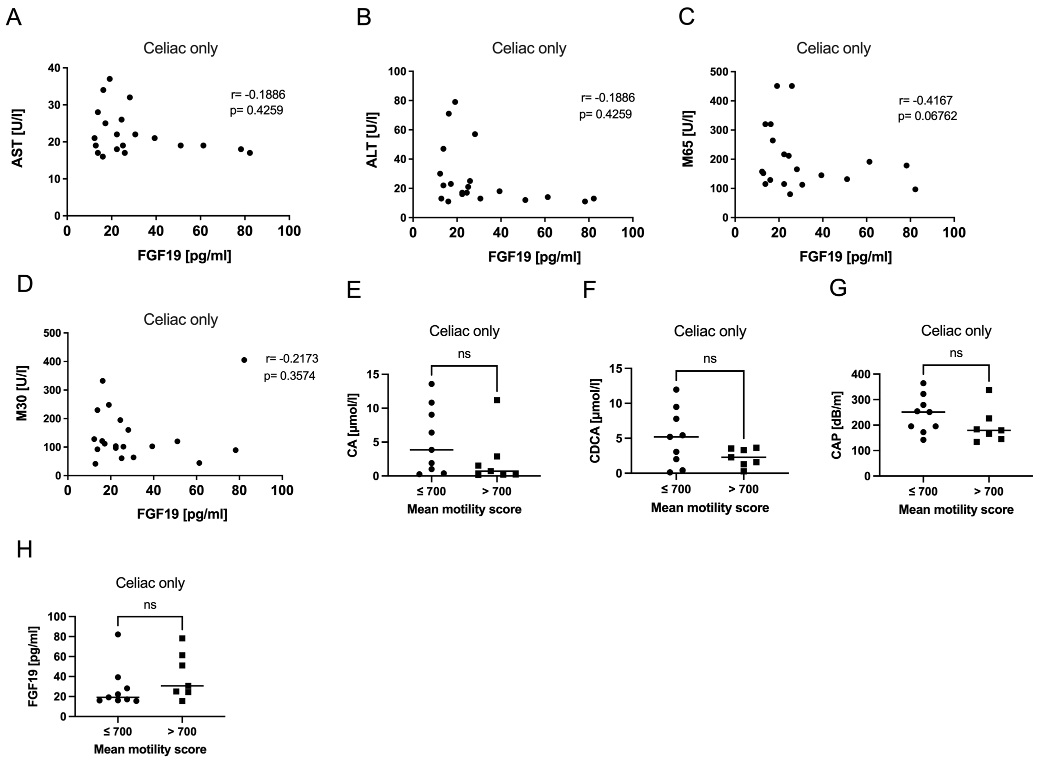

Supplementary Figure S2: Correlation analysis of FGF19 with parameters of liver injury in CeD patients. Serum levels of FGF19 were correlated with liver injury markers AST (A), ALT (B), M65 (C) and M30 (D) in CeD patients. Levels of bile acids CA (E), CDCA (F), CAP (G) and FGF19 (H) were compared in CeD patients with a slower (< 700 mean motility score) versus with a faster gut motility (> 700 mean motility score). ns stands for not significant.
